# Seroepidemiology of Human Bocavirus Infection in Jamaica

**DOI:** 10.1371/journal.pone.0038206

**Published:** 2012-05-29

**Authors:** Joshua W. Hustedt, Celia Christie, Madison M. Hustedt, Daina Esposito, Marietta Vazquez

**Affiliations:** 1 Division of Infectious Diseases, Department of Pediatrics, Yale University School of Medicine, New Haven, Connecticut, United States of America; 2 Department of Pediatrics, University of the West Indies, Kingston, Jamaica; Naval Medical Research Unit 6, United States of America

## Abstract

**Background:**

Human bocavirus (HBoV) is a newly identified human parvovirus. HBoV is associated with upper and lower respiratory tract infections and gastroenteritis in children. Little is known about the seroepidemiology of HBoV in populations in the Caribbean.

**Methods:**

In a cross-sectional study conducted at the University Hospital of the West Indies in Kingston, Jamaica, 287 blood samples were collected from pediatric patients and tested for the presence of HBoV-specific antibody using a virus-like-particle based enzyme-linked immunosorbent assay (ELISA).

**Results:**

HBoV-specific antibodies were found to be present in 220/287 (76.7%) of samples collected from the pediatric population. Seroprevalence of HBoV was highest in those ≥2 years old. The seroepidemiological profile suggests that most children are exposed to HBoV during the first two years of life in Jamaica.

**Conclusion:**

HBoV infection is common in children in Jamaica. HBoV seroprevalence rates in the Caribbean are similar to those previously reported in other areas of the world.

## Introduction

Lower respiratory tract infections, commonly caused by viruses, are one of the leading causes of morbidity and mortality in children worldwide [1]. In the United States, approximately 30,000 children each year are hospitalized during their first year of life due to viral lower respiratory tract infections [2]. In developing countries, the problem is even larger, where up to 1.8 million children die each year due to acute respiratory illnesses [3].

Until recently, most viral lower respiratory infections in children were attributed to respiratory syncytial virus (RSV), parainfluenzavirus, adenovirus, and influenza viruses [4], [5]. Yet, while the etiology of a majority of lower respiratory tract disease is thought to be viral, only around 40% of cases have an etiologically identifiable agent [6], [7], [8]. Over the last 10 years, advances in molecular detection and genomic amplification have led to the discovery of many new viruses including: human metapneumovirus (hMPV) [9]; human coronaviruses: NL63 [10] and HKU1 [11]; SARS-CoV [12], KI and WU polyomaviruses [13], [14]; and human bocavirus (HBoV) [15].

Herein we examine a novel respiratory virus, HBoV; a parvovirus first described in 2005 by Allander *et al.* that has been found to be associated with respiratory tract disease and gastroenteritis in children [15]. Up to 4 different types of HBoV have been isolated, with HBoV1 infecting primarily respiratory epithelium and HBoV2-4 infecting the gastrointestinal tract [16], [17], [18]. Clinical features associated with the detection of HBoV1 infection include fever, rhinorrhea, cough, wheezing, and gastroenteritis [16], [17], [18], [19], [20].

In the United States, HBoV viral DNA was detected by PCR in 5.2% of nasopharyngeal swabs from children in New Haven, CT with respiratory tract disease who tested negative for RSV, influenza A and B virus, parainfluenza viruses 1–3, and adenoviruses, and 0% of asymptomatic control subjects, suggesting a possible causal role for the virus in respiratory tract disease [19]. An additional seroepidemiology study in New Haven, CT found that >90% of children had antibody to HBoV by the age of 4 years, suggesting exposure to HBoV is nearly universal in early childhood [21]. Yet, little is known about HBoV in the developing world.

In this study we sought to determine whether HBoV infection was present in a Caribbean population, in an attempt to elucidate possible differences in HBoV epidemiology in tropical resource poor settings. Using a recently developed ELISA for HBoV [21], we examined hospitalized and non-hospitalized children ages 0–18 years presenting for care at the University Hospital of the West Indies, in Kingston, Jamaica between June 23 and August 10, 2009. The purpose of the study was to generate estimates of exposure to HBoV in the Caribbean and to compare hospitalized and non-hospitalized children of differing ages in an attempt to identify sub-populations that might be at risk for increased HBoV exposure.

## Materials and Methods

This study was approved by the Yale University Human Investigation Committee and the University Hospital of the West Indies Investigation Committee. Written informed consent was obtained from parents or guardians on behalf of the minors/children participants involved in the study. A cross-sectional study was conducted at the University Hospital of the West Indies, located in Kingston, Jamaica from June 23, 2009 to August 10, 2009. Jamaica is a developing country with a population of 2.7 million, gross national income per captia of 4,870 USD's and infant mortality rate of 28 per 1000 live births [22]. The University Hospital of the West Indies is the only academic medical center in Jamaica, and serves as the primary public tertiary care center in Jamaica.

Inclusion criteria for the study included all pediatric patients aged 0–18 years of age presenting for care at the university hospital. 287 total serum samples were collected from the pediatric patient population. 118 (41%) samples were collected via finger-stick method, with the use of the Stat Sampler™ collecting system, from children presenting for routine care and vaccination in well child clinics. The remaining samples were collected from the hospital's clinical chemistry laboratory from left-over venipuncture clinical sampling; including 77 (27%) from inpatient pediatric units, 37 (12%) from pediatric subspecialty clinics, 25 (9%) from the neonatal intensive care, 25 (9%) from pediatric trauma, and 5 (2%) from newborn special care. At the time of collection no information on any symptomatology was collected, as the purpose of the study was to screen for HBoV-specific IgG-antibody representative of previous viral exposure. Both finger stick and venous blood sampling was used as both have been shown to have similar IgG rates in ELISAs, as shown by Vejtorp and Leerhoy [23].

After collection, all blood samples were centrifuged at 10,000 RPM for 10 minutes, and blood serum was collected and stored. Samples were stored at the University Hospital of the West Indies at −20 degrees Celsius, prior to being shipped to Yale University School of Medicine.

### Detection of HBoV-specific antibodies in human serum by HBoV Virus Like Particle–based ELISA

An ELISA developed by Kahn *et al.* was used to detect HBoV-specific antibodies, a detailed description of which can be found elsewhere [21]. Briefly, the ELISA uses virus like particles (VLPs) composed of the VP2 gene of HBoV. The VLPs created by Kahn *et al.* have shown high reactivity with human antibodies to HBoV, and therefore are optimal for use in ELISAs. HBoV VLPs were obtained from Dr. Peter Tattersall at Yale University School of Medicine.

The Kahn *et al.* ELISA protocol was optimized for use with the given study samples. First, samples were run at various dilutions to determine the adequate dilution to use in the assay. A dilution of 1∶40 was chosen for its optimal reactivity with HBoV VLPs in the given ELISA. Next, positive and negative controls were identified and compared to samples with known HBoV infection. The identified positive and negative controls were used in each assay. Finally, all 287 samples were run at a 1∶40 dilution and read on an optical reader at an optical density of 450 nm.

In order to determine a cut-off point to classify samples as seropositive or seronegative all OD450 ELISA values were taken and operationally separated into 2 groups by means of a cutoff of 0.150, as previously published by Kahn *et al.* [21] Next, the mean and standard deviation of all values OD450<0.150 were calculated. The cut off point to classify samples as seropositive or seronegative was calculated as the mean of all OD450<0.150 plus three standard deviations. For this calculation, negative OD450 values were set to zero. Based on these calculations, a serum specimen negative for HBoV was defined as having an OD450≤0.234 (at dilution of 1∶40).

### Statistical Analysis

Data were analyzed with the use of SPSS 18.0. Standard errors for proportions were calculated using standard statistical analysis. Subjects with and without serologic evidence of past HBoV infection were compared with respect to hospitalization status and age group using chi-squared tests for categorical variables and t-tests for continuous variables. Multivariate logistic regression was used to determine significance in a final model. For all tests, a two-tailed critical alpha of 0.05 defined statistical significance.

## Results

287 blood samples were collected from pediatric patients aged 0–18 years at the University Hospital of the West Indies in Kingston, Jamaica. The patients had a mean age of 50.13±50.96 months (range of 0–236 months). 134 (46.7%) of the children were male and 153 (53.3%) were female.

**Figure 1:**
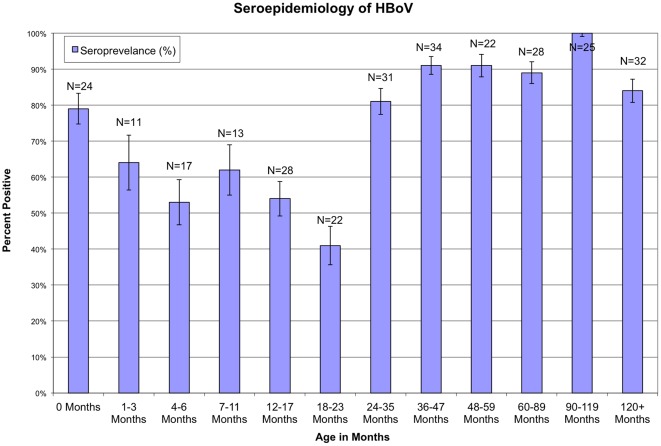
Seroepidemiology of HBoV shown by age group. The no. of serum samples screened and the percentage of seropositive individuals are shown for each age group; bars indicate standard errors.

Overall, 220 (76.7%) of the 287 serum specimens were seropositive for HBoV antibody. Proportion of seropositivity varied by age group (Figure 1). Although 80% of newborns at birth were seropositive for HBoV, this proportion decreased to 50% by six months of age. After six months of age the proportion of seropositive subjects rapidly rebounded, so that by the age of 24 months >80% of children were seropositive. Age was a significant predictor of HBoV exposure in all children (P<0.001), with HBoV seropositive children being older (average age 30.3±17.3 months) than HBoV seronegative children (average age 17.8±12.7 months)

In order to determine if hospitalization was associated with HBoV exposure we characterized sub-groups of 84 hospitalized children from the inpatient pediatrics wards and compared them to 118 non-hospitalized children from the well child clinics (all of whom had never previously been hospitalized). For these comparisons all children from the sub-specialty clinics, neonatal intensive care, pediatric trauma, and special care were excluded on the basis of our inability to determine their previous hospitalization history; and all neonates (<1 month) were also excluded under the premise that a majority of positive values in this age group would represent maternal antibody, not previous HBoV exposure.

Hospitalization proved to be significantly associated with HBoV exposure with 71/84 (84.5%) hospitalized children versus 79/118 (66.9%) of non-hospitalized children testing seropositive to HBoV (P=0.005). However, hospitalized children were significantly older with an average age of 53.2±40.7 months versus 26.2±16.9 months for non-hospitalized children (P<0.001). In a multivariate analysis the age of the subjects proved to be the only statistically significant finding.

## Discussion

Seroepidemiology studies have been conducted in many developed countries to screen for the presence of HBoV, and the virus appears to have a worldwide distribution [19], [24], [25], [26], [27], [28]. To date there are very few reports on HBoV from the developing world [29], [30]. This study is the first seroepidemiology study to show that HBoV antibodies are present in a Caribbean population, with findings that greater than 80% of Jamaican children are exposed to HBoV by the age of 24 months.

The seroepidemiology findings show a high rate of HBoV antibody in the first month of life, with a decline to a nadir around 6 months and a rebound in seropositivity around 24 months. The decline of antibody in infants less than 6 months of age represents waning maternally acquired antibody, as observed in other respiratory viruses such as RSV and human metapneumovirus [31]. This decline overlaps with exposure to HBoV during infancy and early childhood.

Age was the sole predictor associated with the presence of HBoV antibody in the study population. HBoV exposure is common in the population and therefore children have increasing likelihood of exposure as their age increases. We enrolled both healthy and hospitalized children in an effort to both characterize the difference between exposure in these populations and to adequately estimate HBoV seroepidemiology in the overall population. Since HBoV seroepidemiology in subspecialty clinics or hospitalized patients may not be representative of an overall seropositivity rate of the population, we enrolled healthy children in a prospective manner. While seroepidemiology in healthy vs hospitalized children was different, with more hospitalized children being exposed to HBoV than healthy children, this was found to be directly related to the age of the populations. Therefore, since the difference in the hospitalized and healthy children was due to age we feel comfortable in including all the children in the study in the overall estimation of the seroepidemiolgoy of the population.

While HBoV seroepidemiology and prevalence studies have shown evidence for widespread exposure to the virus, the causative role of HBoV in respiratory tract disease is still under investigation. Definitive evidence is difficult to obtain without an *in vitro* culture system and animal model. While there has been one recent successful report of propagation of HBoV in cell culture, this technique has proven difficult to replicate [32]. In the absence of definitive evidence, comparative studies have been used to suggest a causative role of HBoV in acute infection. However, these studies are complicated by the fact that HBoV is often co-detected with other respiratory viruses, which makes it difficult to draw conclusions about its pathogenicity.

One study by Allander *et al.* showed that 5% of symptomatic patients with acute wheezing tested positive for HBoV DNA by PCR in the absence of 12 other viruses known to cause respiratory disease [33]. This suggests a possible causative role for HBoV in acute wheezing. Three other studies, by Kesebir, Fry and Maggi, have also tried to address whether HBoV infection was associated with respiratory tract symptoms. These studies reported that HBoV DNA was more frequently found in patients with acute respiratory tract symptoms than asymptomatic controls [19], [30], [34].

Limitations of our study included lack of serial sampling, lack of detection of viral DNA by PCR, and our inability to verify our ELISA findings with a neutralization assay. Currently, one of the major obstacles to studying the epidemiology and biology of HBoV is the lack of reagents, specifically infectious virus and monospecific anti-HBoV antibodies [32]. Therefore, we were unable to verify our ELISA with a neutralization assay. Instead, we used accepted statistical methods for correct cut-off values in ELISAs [21]. Regrettably, our study was performed prior to the discovery that HBoV has specific subtypes. The ELISA used is not specific to individual HBoV subtypes but rather to HBoV-specific antibodies in general, making the findings not a statement specifically on HBoV1, but of all HBoV subtypes.

In conclusion, this is the first study of HBoV seroepidemiology in a Caribbean population. We showed that HBoV is a common virus infecting children within the first few years of life. Our findings of HBoV seroepidemiology in Jamaica are similar to reports from other areas of the world, reinforcing the notion that HBoV has a worldwide distribution.
